# The relationship between cortical synaptic terminal density marker SV2A and glutamate early in the course of schizophrenia: a multimodal PET and MRS imaging study

**DOI:** 10.1038/s41398-025-03269-8

**Published:** 2025-03-01

**Authors:** Ellis Chika Onwordi, Thomas Whitehurst, Ekaterina Shatalina, Richard Carr, Ayla Mansur, Atheeshaan Arumuham, Martin Osugo, Tiago Reis Marques, Sameer Jauhar, Susham Gupta, Sofia Pappa, Ravi Mehrotra, Maja Ranger, Nikola Rahaman, Eugenii A. Rabiner, Roger N. Gunn, Sridhar Natesan, Oliver D. Howes

**Affiliations:** 1 Institute of Clinical Sciences (ICS), Faculty of Medicine, Imperial College London, London, UK; 2 Psychiatric Imaging Group, Medical Research Council, London Institute of Medical Sciences, Hammersmith Hospital, London, UK; 3 Department of Psychosis Studies, Institute of Psychiatry, Psychology & Neuroscience, King’s College London, London, UK; 4 Centre for Psychiatry and Mental Health, Wolfson Institute of Population Health, Queen Mary University of London, London, UK; 5 East London NHS Foundation Trust, London, UK; 6 City & Hackney Early and Quick Intervention in Psychosis, East London NHS Foundation Trust, London, UK; 7 South London and Maudsley NHS Foundation Trust, London, UK; 8 IQVIA, London, UK; 9 Department of Psychological Medicine, Institute of Psychiatry, Psychology, and Neuroscience, King’s College, London, UK; 10 Tower Hamlets Early Intervention Service, 51 Three Colts Lane, Bethnal Green, East London NHS Foundation Trust, London, UK; 11 Department of Brain Sciences, Imperial College London, The Commonwealth Building, Hammersmith Hospital, London, UK; 12 Research and Development Department, West London NHS Trust, London, UK; 13 Lakeside Unit, West Middlesex University Hospital, West London NHS Trust, London, UK; 14 Westminster Community Rehabilitation Team & Bluebell Lodge, Central and North West London NHS Foundation Trust, London, UK; 15 Westminster and Kensington and Chelsea Early Intervention Service, Central and North West London NHS Foundation Trust, Hathaway House, London, UK; 16 Invicro, Burlington Danes Building, London, UK; 17 Centre for Neuroimaging Sciences, Institute of Psychiatry, Psychology and Neuroscience, King’s College London, London, UK

**Keywords:** Molecular neuroscience, Schizophrenia

## Abstract

Loss of glutamatergic terminals is hypothesised to contribute to excitation-inhibition imbalance in schizophrenia, supported by evidence that the normal positive association between glutamate concentrations and synaptic terminal density is not found in patients with chronic schizophrenia. However, it is unknown whether the relationship between synaptic terminal density and glutamate levels is altered early in the course of illness. To address this, we investigated [^11^C]UCB-J distribution volume ratio (DVR) and glutamatergic markers in healthy volunteers (HV) and in antipsychotic-naïve/free patients with schizophrenia (SCZ) recruited from first-episode psychosis services. Forty volunteers (HV *n* = 19, SCZ *n* = 21) underwent [^11^C]UCB-J positron emission tomography and proton magnetic resonance spectroscopy (^1^H-MRS) imaging in the anterior cingulate cortex (ACC) and left hippocampus to index [^11^C]UCB-J DVR and creatine-scaled glutamate (Glu/Cr) and glutamate in combination with glutamine (Glx/Cr). In the HV but not SCZ group, [^11^C]UCB-J DVR was significantly positively associated with Glu/Cr (Spearman’s rho = 0.55, *p* = 0.02) and Glx/Cr (Spearman’s rho = 0.73, *p* = 0.0004) in the ACC, and with Glu/Cr in the left hippocampus (Spearman’s rho = 0.77, *p* = 0.0001). DVR was significantly lower in the ACC in the SCZ group compared to the HV group (Kolmogorov-Smirnov Z = 1.44, *p* = 0.03). Together, these findings indicate that the normal relationship between levels of a synaptic terminal density marker and levels of glutamate is disrupted early in the course of schizophrenia. This is consistent with the hypothesis that there is loss of glutamatergic terminals at illness onset.

## Introduction

Multiple lines of evidence indicate the involvement of glutamatergic dysfunction in the anterior cingulate cortex (ACC) and hippocampus in the pathophysiology of schizophrenia [1–5]. Meta-analyses of magnetic resonance spectroscopy (MRS) studies have shown lower glutamate levels in medial frontal cortical regions including the ACC [6] and greater levels of Glx (glutamate in combination with glutamine) in medial temporal regions including the hippocampus [7] in patients with schizophrenia compared to controls. It has been hypothesised that there are lower levels of glutamatergic terminals in schizophrenia [8, 9]. This hypothesis was tested recently in a study that combined glutamate MRS with [^11^C]UCB-J PET, which indexes synaptic vesicle glycoprotein 2 A (SV2A), an in vivo marker of synaptic terminal density [10]. This study showed that glutamate levels were significantly positively correlated to synaptic terminal density in the ACC and left hippocampus in healthy controls, but not in patients with schizophrenia [10]. The healthy control findings are consistent with prior ex vivo findings that a large proportion of synapses in the mammalian brain are glutamatergic [11–13]. The schizophrenia sample in that study consisted of patients with chronic schizophrenia who were taking antipsychotic medication [10], who showed evidence for lower synaptic terminal density in vivo in the ACC and hippocampus relative to controls with large effect sizes [14]. However, studies of synaptic terminal density in patients early in the course of schizophrenia compared to controls yield mixed findings, with one study finding similarly large differences [15], but another study indicating effects may not be as marked [16]. To our knowledge, no previous study has explored the link between synaptic terminal density and glutamate levels early in the course of schizophrenia in patients free from antipsychotic treatment. Thus, it remains unknown whether patients early in the course of illness and free from antipsychotic medication also show a loss of the normal, positive relationship between synaptic terminal density and glutamate, or whether this develops later during illness.

Therefore, we conducted a multimodal [^11^C]UCB-J PET and ^1^H-MRS imaging study to test the relationship between the synaptic terminal marker SV2A and glutamate levels in unmedicated patients with schizophrenia within the first episodes of psychosis. Previous work has not identified a significant association between antipsychotic drug exposure and [^11^C]UCB-J binding [14, 15, 17], and antipsychotics are not reported to bind directly to SV2A [18]. Therefore, we anticipated that findings would be similar to those in the study of antipsychotic-treated patients [10], thus hypothesising a significant positive relationship between SV2A and glutamate levels in healthy volunteers, but not in antipsychotic-free or -naïve patients early in the course of schizophrenia.

## Materials and methods

The study protocol was approved by the London-West London & GTAC Research Ethics Committee, United Kingdom (reference: 16/LO/1941). The Administration of Radioactive Substances Advisory Committee, United Kingdom, approved the administration of radioactive material. We obtained written informed consent from all volunteers before their participation in the study, which was conducted in accordance with the Declaration of Helsinki (1996).

We recruited 40 subjects to this study (19 healthy volunteers [HVs] and 21 patients with schizophrenia [SCZ]). The MRS data for the SCZ group have not been reported before. The MRS data for 15 healthy volunteers and PET data for all volunteers have been reported previously [10, 16]. Inclusion criteria for all volunteers were: capacity to consent, 18-65 years of age, and a having normal blood coagulation test result to facilitate arterial blood sampling.

We recruited patients from London first-episode psychosis services. Inclusion criteria for patients with SCZ were: meeting DSM-5 criteria [19] for SCZ and being antipsychotic-naïve or free from antipsychotic medication for at least 4 weeks or five half-lives prior to [^11^C]UCB-J imaging, whichever was longer, to ensure adequate antipsychotic elimination [20]. HVs were required to have no history of a mental disorder or family history of SCZ. Exclusion criteria for all patients and healthy volunteers were: history of head trauma resulting in a loss of consciousness; drug or alcohol dependence (except nicotine dependence); neurological disorder; significant medical disorder; taking drugs known to interact with SV2A (e.g. levetiracetam, brivaracetam, loratadine or quinine [21]); or contraindications to imaging. Age, gender, smoking and cannabis use status were recorded.

### Clinical assessments

Diagnosis was confirmed by a research clinician using the Structured Clinical Interview for DSM-5 [22]. We determined illness duration as the time from first psychotic symptoms for each patient [23]. We measured symptom severity using the Positive and Negative Syndrome Scale (PANSS) [24]. We screened healthy volunteers using the Structured Clinical Interview for DSM-5 to exclude psychiatric illness and family history of psychosis.

### Magnetic resonance imaging

Each subject underwent structural magnetic resonance imaging (MRI) to enable delineation of anatomical regions of interest (ROIs) and to guide placement of MRS voxels. We acquired T1-weighted three-dimension magnetisation prepared rapid acquisition gradient echo images using a Siemens Magnetom Prisma 3T scanner (Siemens, Erlangen, Germany) according to the following parameters: echo time = 2.28 ms, repetition time = 2300.0 ms, flip angle = 9°, field of view = 256 × 256 mm, 176 sagittal slices of 1 mm thickness, voxel size = 1.0 × 1.0 × 1.0 mm.

### ^1^H-MRS acquisition

**Fig. 1:**
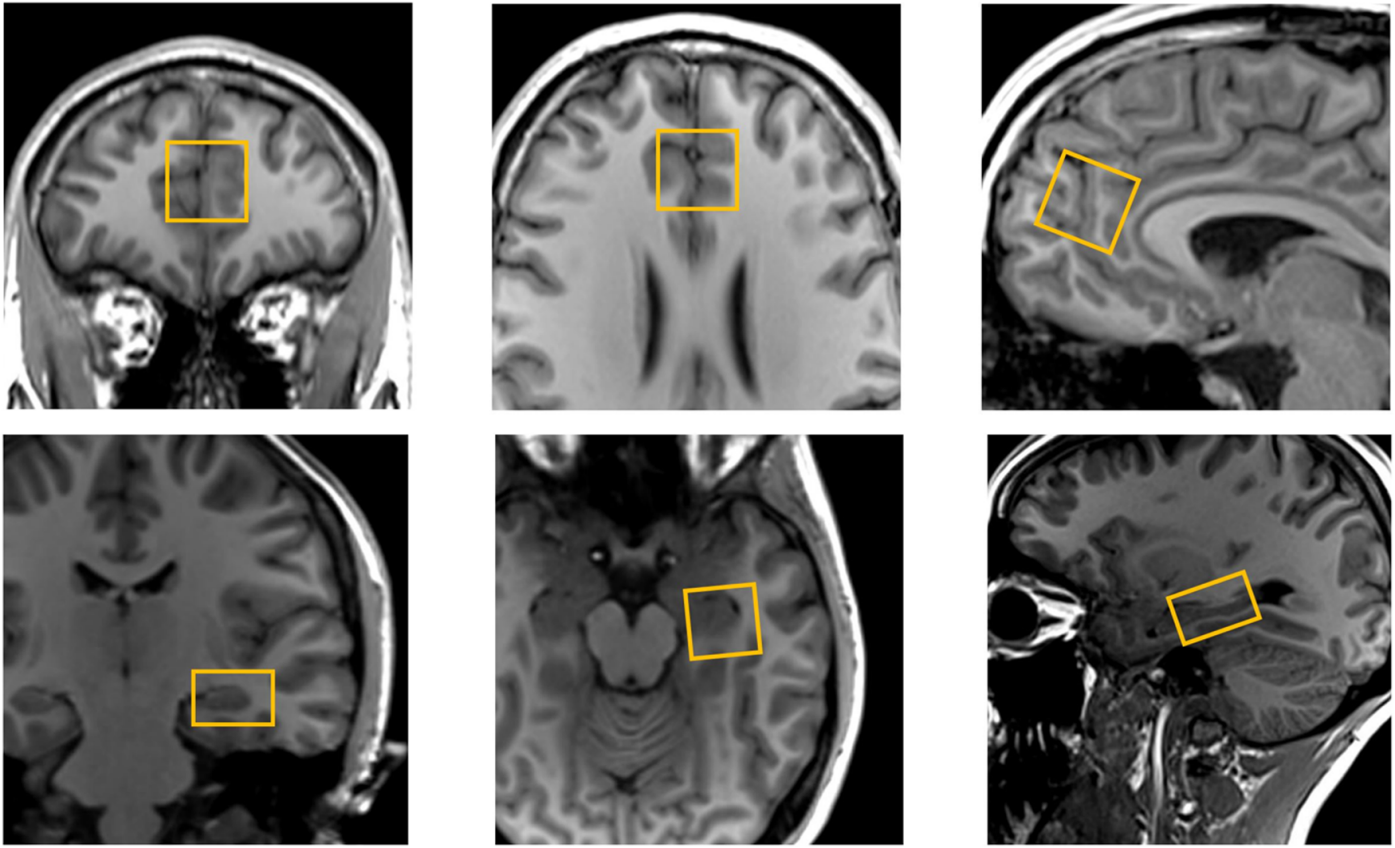
^1^H-MRS voxel position in the anterior cingulate cortex (top panel) and left hippocampus (bottom panel).

We conducted single voxel ^1^H-MRS as reported previously [10]. We used the Point RESolved technique (echo time = 30 ms, repetition time = 3000 ms, 96 averages, Vector size = 2048, Bandwidth = 2500 Hz). Before MRS acquisition, the B0 homogeneity across the voxel was automatically optimised then manually fine-tuned with the first-order shim gradients to achieve a water linewidth $\le$ 18 Hz. A 20 × 20 × 20 mm voxel was placed in the ACC, immediately anterior to the genu of the corpus callosum in the midline (Fig. 1). A 20 × 20 × 15 mm voxel was placed in the left hippocampus, angled parallel to the anterior horn of the temporal lobe, placed just posterior to the amygdala, avoiding the petrous bones (Fig. 1).

#### PET acquisition

##### PET imaging

Subjects received a low-dose computed tomography scan for attenuation and scatter correction, followed by an [^11^C]UCB-J microdose ( ≤ 300 MBq) delivered as a smooth bolus injection via an intravenous cannula over 20 seconds. We acquired PET data for 90 minutes using a Biograph 6 HiRez PET-CT scanner (Siemens).

##### Arterial blood sampling

We collected radial arterial blood samples throughout the PET scan to measure the arterial input function as has been detailed elsewhere [25]. Briefly, we used a continuous automatic blood sampling system to measure whole blood activity for the first 15 minutes (Allogg AB, Mariefred, Sweden), and took discrete samples 10, 15, 20, 25, 30, 40, 50, 60, 70, 80 and 90 minutes after tracer injection. We used a Perkin Elmer 1470 10-well gamma counter to measure plasma radioactivity and total blood concentrations, and high-performance liquid chromatography to measure the plasma radioactivity fraction constituted by unchanged parent radioligand from discrete blood samples. We used ultrafiltration in triplicate to measure the [^11^C]UCB-J plasma free fraction from an arterial blood sample taken before tracer injection.

##### Image analysis

Consistent with our prior study investigating the relationship between synaptic terminal density and glutamate [10], we used distribution volume ratio (DVR) as our primary [^11^C]UCB-J outcome measure. This is because DVR uses a reference region approach to adjust for nonspecific tracer uptake, thereby reflecting more closely the signal specific to SV2A in the ROI than volume of distribution (*V*_T_) [26], which indexes the radioligand concentration specifically bound to SV2A as well as the nondisplaceable uptake. Moreover, [^11^C]UCB-J *V*_T_ values show greater variability than DVR values [25] and so likely have decreased sensitivity to group differences [27], as found in previous [^11^C]UCB-J analyses [14, 16].

We undertook processing and modelling using MIAKAT version 4.3.7 (http://www.miakat.org/MIAKAT2/index.html), implemented in MATLAB (version R2018b; The MathWorks, Inc.) with functions from FSL (version 5.0.10; FMRIB) and SPM12 (Wellcome Trust Centre for Neuroimaging, http://www.fil.ion.ucl.ac.uk/spm).

Each subject’s MRI underwent brain extraction using FSL and gray matter segmentation and rigid-body coregistration to a standard reference space [28] using SPM12 as implemented via MIAKAT. The template brain image and associated Clinical Imaging Centre atlas [29] were then warped nonlinearly to the subject’s MRI. We used the ACC and left hippocampus as defined on this atlas as our primary ROIs, consistent with the approach taken in the prior study in chronic patients [10]. We generated the centrum semiovale (CS) ROI from the automated anatomical labelling template [30] according to parameters defined for its use as a reference region to estimate nondisplaceable [^11^C]UCB-J binding [31].

For each subject, individual PET images were corrected for motion through frame-to-frame rigid-body registration using the 14th frame (acquired 9–11 minutes after injection) as the reference frame. The summed PET image was co-registered to the MRI. We generated time activity curves for each ROI.

Arterial input function and regional time activity curve data were analysed together using the 1-tissue compartment model, which produces reliable [^11^C]UCB-J *V*_T_ estimates [25, 32]. Grey matter masks were applied to ROIs within MIAKAT to extract regional grey matter *V*_T_. Regional DVR was obtained using the CS as a pseudoreference region [25, 31], thereby deriving DVR as a ratio of ROI *V*_T_ to CS *V*_T_.

MRS data were analysed with LC Model® 6.3–1 L for automatic quantification of in vivo ^1^H-MR spectra [33] to index concentrations of glutamate (Glu) and Glx. Neurometabolite values were scaled to creatine (Cr), which provides an internal reference. We report Glu/Cr and Glx/Cr levels as our neurometabolite outcome measures. Metabolite analyses were restricted to spectra with Cramér-Rao bounds ≤20% and signal-to-noise ratio ≥5%.

##### Sample size and power calculation

We determined the minimum sample size needed to test our primary hypothesis using G∗power version 3.1.9.3 (https://www.psychologie.hhu.de/arbeitsgruppen/allgemeine-psychologie-und-arbeitspsychologie/gpower). Our previous study identified significant associations between [^11^C]UCB-J DVR and glutamatergic measures with a mean Pearson’s correlation coefficient of 0.6 in control participants [10]. The power calculation indicated that a minimum sample size of 19 subjects per group would have more than 80% power to detect a significant association between [^11^C]UCB-J binding and glutamatergic measures with *r* ≥ 0.6.

##### Statistical analysis

We conducted statistical analyses using IBM SPSS Statistics, Version 25, and RStudio Version 1.4.1106 (RStudio Team (2021), RStudio, Inc., Boston, MA (http://www.rstudio.com/)). We used the Shapiro-Wilk test to evaluate normality of distribution. Our primary analysis tested the relationship between grey matter [^11^C]UCB-J DVR and Glu/Cr in the ACC and left hippocampus in HV and SCZ groups. We also conducted exploratory analyses testing the relationship between grey matter [^11^C]UCB-J DVR and Glx/Cr. Where data were normally distributed, we tested relationships between variables using Pearson product-moment correlation, and where data were not normally distributed, we used Spearman’s rho.

Group differences in clinical and demographic variables, *V*_T_ in the CS, and DVR and neurometabolite levels in the ACC and left hippocampus were assessed using two-tailed independent sample *t* tests for normally distributed data, Kolmogorov–Smirnov tests for non-normally distributed data and Chi-squared tests for categorical data.

## Results

Forty participants (HV *n* = 19 [16 male and 3 female]; SCZ *n* = 21 [17 male and 4 female]) completed the study. The groups were well matched in terms of age, sex, ethnicity, proportion of current smokers and cannabis users within the last month, and there were no significant group differences in PET imaging characteristics, [^11^C]UCB-J injected activity, injected cold mass, specific radioactivity, minimum purity, plasma-free fraction (*f*_p_) or centrum semiovale *V*_T_ (see Table 1).

**Table 1:** Clinico-demographic and imaging variables in healthy volunteer (HV) and schizophrenia (SCZ) groups.

	HV	SCZ	*t*	Kolmogorov–Smirnov *Z*	Chi-squared	df	*p*
Age (years)	29.79 [1.84, 8.03]	26.52 [1.74, 7.99]	–	1.01	–	–	0.26
Male, female (*n*)	16, 3	17, 4	–	–	0.07	–	0.79
Ethnicity White, Black, Asian, other (*n*)	10, 5, 3, 1	4, 11, 4, 2	–	–	5.21	–	0.16
Current smoker (*n*)	5	7	–	–	0.23	–	0.63
Cannabis users within last month (*n*)	1	2	–	–	0.26	–	0.61
Activity injected (MBq)	265.42 [5.10, 22.22]	221.24 [12.84, 58.85]	–	1.23	–	–	0.10
Injected mass (μg)	3.19 [0.24, 1.04]	3.18 [0.35, 1.59]	–	0.56	–	–	0.91
Specific radioactivity (GBq/μmol)	30.17 [2.76, 12.03]	25.64 [2.21, 10.11]	–	0.67	–	–	0.77
Minimum purity (fraction)	99.97 [0.03, 0.15]	100 [0.00, 0.00]	–	0.17	–	–	1.00
[^11^C]UCB-J plasma-free fraction (*f*_p_)	0.24 [0.006, 0.02]	0.27 [0.007, 0.03]	–	1.20	–	–	0.12
Illness duration (years)	–	2.67 [0.46, 2.11]	–	–	–	–	–
Drug-free interval (days)	–	180.42 [27.71, 120.78]	–	–	–	–	–
PANSS Total score	–	65.29 [3.29, 15.09]	–	–	–	–	–
PANSS Positive score	–	17.05 [1.24, 5.69]	–	–	–	–	–
PANSS Negative score	–	17.81 [0.95, 4.33]	–	–	–	–	–
PANSS General score	–	30.43 [1.81, 8.32]	–	–	–	–	–
CS *V*_T_	5.53 [0.14, 0.62]	6.34 [0.37, 1.69]	–	0.97	–	–	0.30
**ACC**							
DVR	4.09 [0.12, 0.52]	3.66 [0.14, 0.66]	–	1.44	–	–	0.03
Glu/Cr	1.14 [0.02, 0.07]	1.17 [0.02, 0.10]	–	1.29	–	–	0.07
Glx/Cr	1.34 [0.03, 0.12]	1.35 [0.04, 0.18]	0.27	–	–	38	0.79
**Left hippocampus**							
DVR	2.75 [0.09, 0.38]	2.57 [0.11, 0.49]	–	0.63	–	–	0.83
Glu/Cr	1.01 [0.04, 0.19]	1.06 [0.03, 0.13]	0.11	–	–	38	0.30
Glx/Cr	1.42 [0.07, 0.30]	1.42 [0.04, 0.19]	0.05	–	–	38	0.96

Values are mean and standard error of the mean, standard deviation (SEM, SD) or, where indicated, number (n).

In the SCZ group, no patients were taking antipsychotic medication during the study. Two patients were antipsychotic-naïve, and 19 had previously taken antipsychotic medication prior to their involvement in the study (mean [SEM, SD] interval of 180.42 (27.71, 120.78) days, minimum of 41 days between [^11^C]UCB-J imaging and most recent antipsychotic drug exposure). No patients had comorbid DSM-5 psychiatric diagnoses. Injected mass (*p* = 0.009), plasma free fraction (*p* = 0.007), ACC Glu/Cr (*p* = 0.02), CS *V*_T_ (*p* < 0.001), ACC DVR (*p* = 0.01), and left hippocampal DVR (*p* = 0.006) were not normally distributed in SCZ group. Specific radioactivity (*p* = 0.04) and minimum purity fraction (*p* < 0.001) were not normally distributed in the HV group. Age (*p* = 0.001 in HV, *p* = 0.04 in SCZ) was not normally distributed in either group.

### The relationship between [^11^C]UCB-J DVR and glutamatergic measures in the ACC

**Fig. 2:**
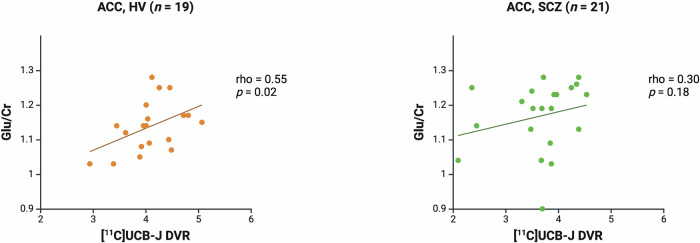
[^11^C]UCB-J distribution volume ratio (DVR) and levels of glutamate (Glu/Cr) in the anterior cingulate cortex (ACC). Significant positive relationship between [^11^C]UCB-J DVR and Glu/Cr levels in the ACC in the healthy volunteer group (HV, rho = 0.55, *p* = 0.02), and no significant relationship in the schizophrenia group (SCZ, rho = 0.30, *p* = 0.18). Linear regression line shown.

There was a significant positive correlation between [^11^C]UCB-J DVR and Glu/Cr in the ACC in the HV (Spearman’s rho = 0.55, *p* = 0.02) but not SCZ group (Spearman’s rho = 0.30, *p* = 0.18, Fig. 2). A significant positive association between [^11^C]UCB-J DVR and Glx/Cr was also observed in the HV (Spearman’s rho = 0.73, *p* = 0.0004) but not in the SCZ group (Spearman’s rho = 0.36, *p* = 0.11; Supplementary Fig. 1). Post-hoc analysis showed that there was no significant difference between the HV and SCZ groups in the strength of the bivariate [^11^C]UCB-J DVR-Glu/Cr correlation (Fisher’s r-to-z: *z* = 0.90, *p* = 0.37) or [^11^C]UCB-J DVR-Glx/Cr correlation (*z* = 1.61, *p* = 0.11). Mean (SEM, SD) ACC [^11^C]UCB-J DVR was significantly lower in the SCZ (3.66 [0.14, 0.66]) compared to the HV group (4.09 [0.12, 0.52], Kolmogorov-Smirnov Z = 1.44, *p* = 0.03). There were no significant differences between groups in Glu/Cr or Glx/Cr levels in the ACC (Table 1).

### The relationship between [^11^C]UCB-J DVR and glutamatergic measures in the left hippocampus

**Fig. 3:**
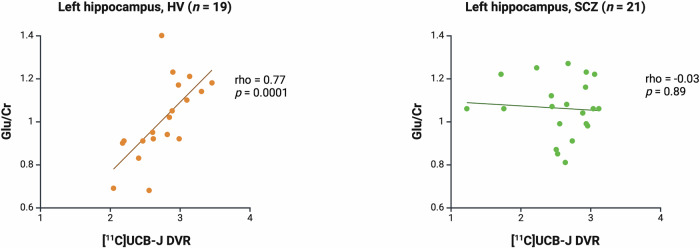
[^11^C]UCB-J distribution volume ratio (DVR) and levels of glutamate (Glu/Cr) in the left hippocampus. Significant positive relationship between [^11^C]UCB-J DVR and Glu/Cr levels in the left hippocampus in the healthy volunteer group (HV, Spearman’s rho = 0.77, *p* = 0.0001) and no significant relationship in the schizophrenia group (SCZ, rho = −0.03, *p* = 0.89). Linear regression line shown.

There was a significant positive association between [^11^C]UCB-J and Glu/Cr in the left hippocampus in the HV (Spearman’s rho = 0.77, *p* = 0.0001) but not SCZ group (Spearman’s rho = −0.03, *p* = 0.89, Fig. 3). There was no significant association between [^11^C]UCB-J DVR and Glx/Cr in the HV (Spearman’s rho = 0.38, *p* = 0.11) or SCZ group (Spearman’s rho = −0.30, *p* = 0.19, Supplementary Fig. 2). Post-hoc analysis showed that there was a significant difference between the HV and SCZ groups in the strength of the bivariate [^11^C]UCB-J DVR-Glu/Cr correlation (*z* = 3.06, *p* = 0.002) and [^11^C]UCB-J DVR-Glx/Cr correlation (*z* = 2.07, *p* = 0.04). There were no significant group differences in [^11^C]UCB-J DVR, Glu/Cr or Glx/Cr levels in the left hippocampus (Table 1).

### Associations between symptom severity and imaging measures

There were no significant associations between PANSS scores and DVR, Glu/Cr or Glx/Cr in the ACC or left hippocampus (see Supplementary Table 1).

## Discussion

We found significant positive correlations between a synaptic terminal density marker and glutamatergic markers in both the ACC and hippocampus in healthy volunteers but not in patients early in the course of schizophrenia. These findings are consistent with our previous findings in healthy volunteers and patients with chronic schizophrenia [10].

Our findings have implications for understanding the pathophysiology of schizophrenia. Taken together with findings that synaptic terminal density is markedly reduced in chronic schizophrenia [14, 17], but possibly only subtly lower early in the course of illness [16], these findings suggest that the deficits in synaptic terminal marker levels reported in schizophrenia are driven by a loss of glutamatergic terminals and/or a reduced glutamatergic-GABAergic terminal ratio early in the course of schizophrenia, which progresses over time. In normal development, post-pubertal synaptic elimination particularly affects glutamatergic synapses [34, 35], facilitating the balance between excitation and inhibition which optimises signal-to-noise ratio in cortical neuronal arrays [36]. Loss of glutamatergic synapses, or of the proportion of synapses that are glutamatergic, may contribute to the excitation-inhibition imbalances and cortical network dysfunction [37–41], contributing to cognitive dysfunction in schizophrenia [42]. The current study, together with our previous findings [10], suggests a disease-related synaptic terminal loss affecting glutamatergic synapses in particular. Future development of in vivo molecular imaging probes specific to glutamatergic synapses would enable direct verification. Nonetheless, our findings may suggest that cortical glutamatergic synaptic terminal density is a potential target in the treatment of schizophrenia.

DVR was significantly lower in the ACC in the SCZ group, consistent with previous reports in chronic schizophrenia [14, 17], and in keeping with *V*_T_ findings when corrected for *f*_p_ in a largely overlapping sample [16]. This finding contrasts with our results in our prior analysis of an overlapping sample, where we found a non-significant trend towards lower DVR in the early-course schizophrenia group compared to healthy volunteers (*p* = 0.09) [16]. The sample reported in the current analysis excludes two healthy volunteers reported in the prior study, for whom MRS data were not captured. Given the lower sample size in the healthy volunteer group in this study, our finding of lower DVR in the ACC in the schizophrenia group should be interpreted cautiously. We found no significant difference in DVR in the left hippocampus, in keeping with previous findings in the left hippocampus in chronic schizophrenia [10]. This is in contrast to the large reductions in [^11^C]UCB-J binding identified in another early-course schizophrenia group relative to controls [15], but it is important to note that the current study has a significantly greater number of healthy volunteers and patients, and so may have greater power to test this question. Our finding also contrasts with those from studies of chronic schizophrenia, which identified large reductions in [^11^C]UCB-J binding in hippocampal regions of interest incorporating both hippocampi [14, 17], whereas here we investigated the left hippocampus early in the course of illness. Thus, the discrepancy between our current and previous findings may be driven by chronicity of illness, and/or disease effects in the right hippocampus not captured by our current analysis. In contrast with the findings from meta-analyses of ^1^H-MRS studies [6, 7], we did not identify significant differences in Glu/Cr or Glx/Cr between groups. However, this is consistent with several individual case-control studies of a similar size [43–46]. This difference may reflect a lack of power in our present study to detect the small effects in ACC glutamate levels reported in meta-analyses.

### Strengths and limitations

To our knowledge, this is the first study exploring the relationship between a synaptic terminal density marker and glutamate levels in vivo early in the course of schizophrenia. Previous work has shown that brain glutamate concentrations are greatest in glutamatergic terminals, and significantly lower in glial and extracellular compartments [47]. In addition, neurotransmitter and metabolic pools of glutamate are tightly coupled [48], and synaptic vesicle density and glutamate immunostaining intensity are significantly positively correlated in rats [49]. This evidence, when taken together with our finding that SV2A and glutamate levels are positively associated in the healthy human brain, supports the hypothesis that the glutamate signal captured by ^1^H-MRS is related to the presynaptic neurotransmitter pool of glutamate.

None of the patients in this study were taking medication, removing the influence of current medication exposure. However, most had previously taken antipsychotic medication (*n* = 19). It is unclear whether prior antipsychotic exposure influences our findings. However, antipsychotic drugs are not reported to bind directly to SV2A [18]. Moreover, previous work failed to show an effect of haloperidol or olanzapine exposure on SV2A binding in the rat brain ex vivo [14], or an association between levels of antipsychotic drug exposure and [^11^C]UCB-J binding in the human brain in vivo [14, 15, 17]. There is some evidence from longitudinal MRS studies that brain glutamate levels may reduce following the initiation of antipsychotic drug treatment [50], but it remains unclear whether effects persist following the discontinuation of treatment. Thus, current evidence indicates that our findings are unlikely to be confounded by prior antipsychotic exposures, but it would be valuable to explore the relationship between measures of synaptic density and glutamate in larger cohorts of antipsychotic drug-naïve patients with schizophrenia to confirm findings.

A strength of the current study is that groups were well matched for smoking status. In previous work testing the relationship between SV2A and glutamate levels, there were significantly more smokers in the schizophrenia than in the healthy volunteer group [10]. Notably, in that previous study, smoking was associated with lower hippocampal Glu/Cr levels in healthy volunteers, but this did not impact on the strength of the relationship between SV2A and Glu/Cr levels [10]. Moreover, the main findings relating to the relationship between glutamate and SV2A results remain largely consistent between these studies.

There are key considerations regarding our measurement of glutamatergic markers using ^1^H-MRS. At 3T, glutamate and glutamine concentrations are challenging to separate due to substantial spectral overlap [51, 52]. Greater magnetic field strengths are needed to quantify these signals with greater precision. In addition, we used creatine as an internal reference, reporting creatine-corrected glutamatergic levels. Disease effects on creatine levels could affect the interpretation of our findings. However, multiple meta-analyses of ^1^H-MRS studies have failed to identify a significant difference in brain creatine levels in schizophrenia relative to healthy controls [53–55], suggesting our analysis is unlikely to be significantly affected by the use of creatine as an internal reference.

We used the CS, a white matter region containing low levels of SV2A and showing low [^11^C]UCB-J uptake, as a reference region to calculate [^11^C]UCB-J DVR as our PET outcome measure [31]. This approach adjusts for nonspecific binding, providing a signal more specific to SV2A, and more sensitive to schizophrenia-related differences in SV2A levels, than *V*_T_ [14, 16, 17]. However, there are limitations to the use of the CS as a reference region, given that the tissue composition of white matter is distinct from that of grey matter [26], and there is a small amount of displacement of [^11^C]UCB-J in the CS by levetiracetam, an SV2A-specific drug, suggesting a low level of specific binding in this region. A difference in CS *V*_T_ between schizophrenia and healthy control groups could systematically bias our findings and, whilst CS *V*_T_ was numerically greater in the schizophrenia group in this study, this difference was not significant (*p* = 0.30), consistent with separate work [17].

This is a cross-sectional study, so we are unable to determine the mechanistic links between glutamate, SV2A and schizophrenia. Longitudinal combined PET-MRS studies are needed to test whether there is evidence for progressive loss of glutamatergic terminals in schizophrenia, a change in the SV2A-glutamate relationships over time, and possible effects of medication on these relationships, as seen in longitudinal PET-MRS studies of other neurobiological systems in schizophrenia [56].

We found a significant difference between patients and controls in the strength of the [^11^C]UCB-J DVR-Glu/Cr and -Glx/Cr correlations in the left hippocampus, but not in the ACC, using Fisher’s r-to-z transformation test. This study was designed to test SV2A-glutamate relationships within groups, and may be underpowered to test group differences in correlation strengths. Future studies with larger sample sizes are needed to test for differences between healthy volunteer and schizophrenia groups in SV2A-glutamate correlation strength.

## Conclusions

The normal relationship between synaptic terminal density and glutamate levels is disrupted early in the course of schizophrenia in antipsychotic-free patients, extending previous work showing similar deficits in chronic medicated schizophrenia. This may be due to a loss of glutamatergic synaptic terminals and/or a lower proportion of glutamatergic terminals early in the course of schizophrenia, which persists through the course of illness, and which is unaffected by ongoing exposure to antipsychotic medication.

## Supplementary information

The relationship between cortical synaptic terminal density marker SV2A and glutamate early in the course of schizophrenia: a multimodal PET and MRS imaging study - Supplemental material

## Data Availability

Imaging and related clinical data will be made available upon reasonable request.
