# Supplementary material for: The relationship between cortical synaptic terminal density marker SV2A and glutamate early in the course of schizophrenia: a multimodal PET and MRS imaging study
Source: Transl Psychiatry. 2025 Mar 1;15:70. doi: 10.1038/s41398-025-03269-8 (PMC11873237; doi:10.1038/s41398-025-03269-8)
Supplement: Supplementary file 1 — The relationship between cortical synaptic terminal density marker SV2A and glutamate early in the course of schizophrenia: a multimodal PET and MRS imaging study - Supplemental material [file 41398_2025_3269_MOESM1_ESM.docx]

**The relationship between cortical synaptic terminal density marker SV2A and glutamate early in the course of schizophrenia: a multimodal PET and MRS imaging study**

**Supplementary material**

**Authors**

Ellis Chika Onwordi PhD^[[1]](#footnote-1)^^,^^[[2]](#footnote-2)^^,^^[[3]](#footnote-3),^^[[4]](#footnote-4),^^[[5]](#footnote-5)^, Thomas Whitehurst PhD^1,2,3,^^[[6]](#footnote-6)^, Ekaterina Shatalina PhD^1,2,3^, Richard Carr MBChB^2,3,^^[[7]](#footnote-7)^, Ayla Mansur PhD^[[8]](#footnote-8)^, Atheeshaan Arumuham MBChB^1,2,3^, Martin Osugo MBChB^1,2,3^, Tiago Reis Marques PhD^1,2,3^, Sameer Jauhar PhD^[[9]](#footnote-9)^, Susham Gupta FRCPsych^[[10]](#footnote-10)^, Sofia Pappa PhD^[[11]](#footnote-11),^^[[12]](#footnote-12)^, Ravi Mehrotra MRCPsych^[[13]](#footnote-13)^, Maja Ranger MD^[[14]](#footnote-14)^, Nikola Rahaman MRCPsych^[[15]](#footnote-15)^, Eugenii A. Rabiner FRCPsych SA^[[16]](#footnote-16),^^[[17]](#footnote-17)^, Roger N. Gunn PhD^11,16^, Sridhar Natesan PhD^1,2,3^ and Oliver D. Howes PhD^1,2,3^


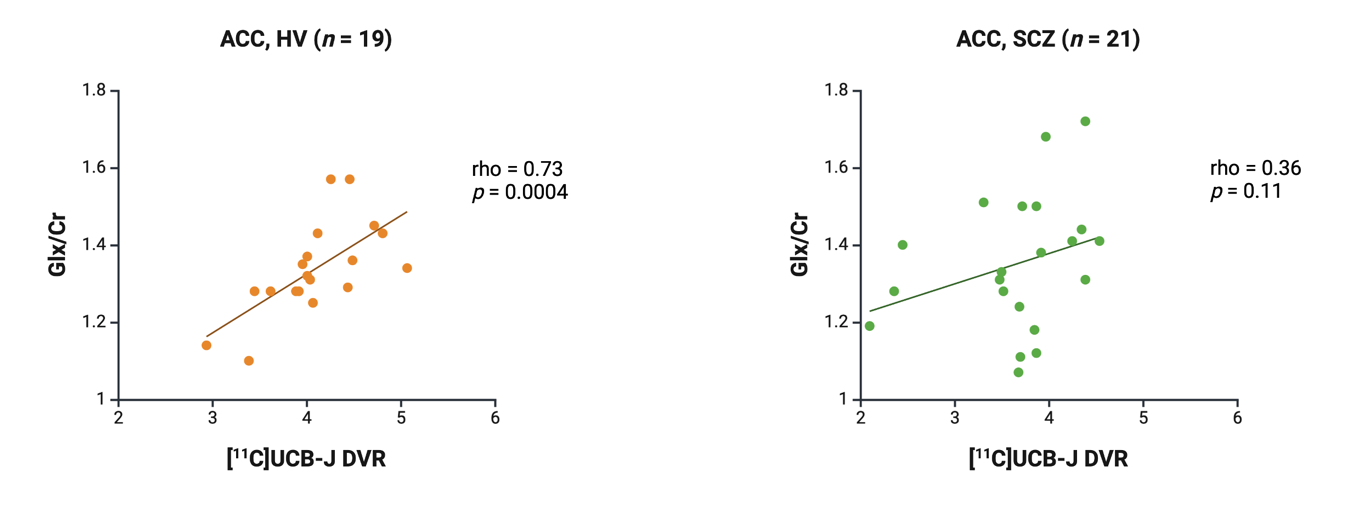


*Supplementary Figure 1 [^11^C]UCB-J distribution volume ratio (DVR) and levels of glutamate combined with glutamine (Glx/Cr) in the anterior cingulate cortex (ACC). Significant positive relationship between [^11^C]UCB-J DVR and Glx/Cr levels in the ACC in the healthy volunteer group (HV, Spearman’s rho = 0.73, p = 0.0004), and no significant relationship in the schizophrenia group (SCZ, rho = 0.36, p = 0.11).*


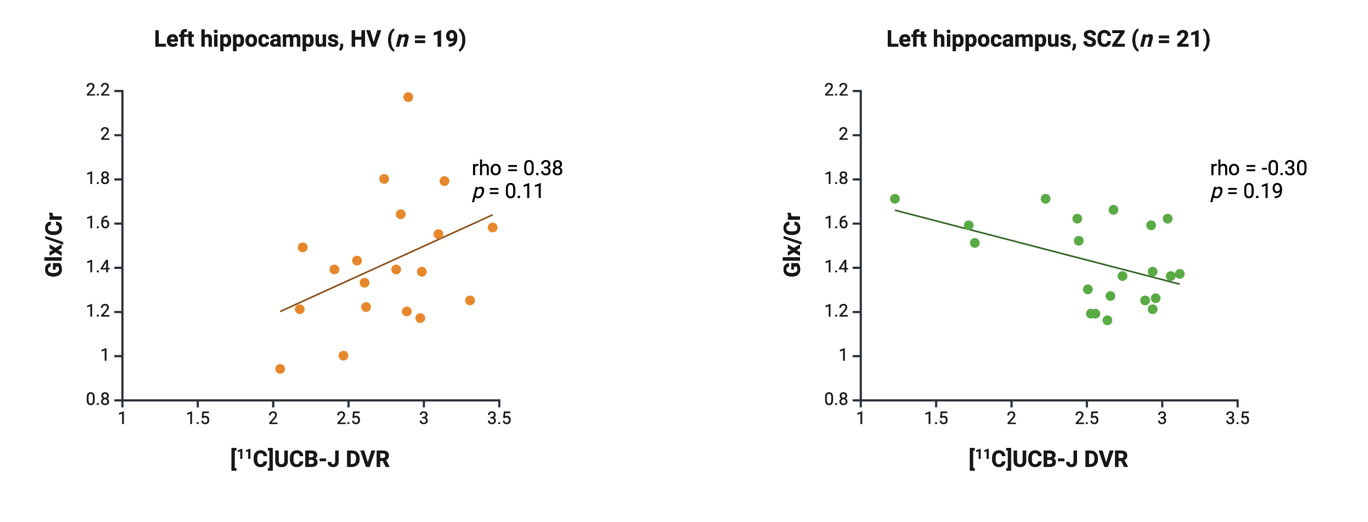


*Supplementary Figure 2 [^11^C]UCB-J distribution volume ratio (DVR) and levels of glutamate combined with glutamine (Glx/Cr) in the left hippocampus. No significant relationship between [^11^C]UCB-J DVR and Glx/Cr levels in the left hippocampus in the healthy volunteer group (HV, Spearman’s rho = 0.38, p = 0.11) or in the schizophrenia group (SCZ, rho = -0.30, p = 0.19).*

*Supplementary Table 1 Associations between PANSS scores (total and positive, negative and general subscale scores) and [11C]UCB-J DVR, Glu/Cr and Glx/Cr in the hippocampus and anterior cingulate cortex, explored in the schizophrenia group (n = 21). There were no significant associations detected. All correlation coefficients reported are Spearman’s rho.*

|  | | PANSS total | | PANSS positive | | PANSS negative | | PANSS general | |
| --- | --- | --- | --- | --- | --- | --- | --- | --- | --- |
|  |  | Correlation coefficient | *P* value | Correlation coefficient | *P* value | Correlation coefficient | *P* value | Correlation coefficient | *P* value |
| ACC | [^11^C]UCB-J DVR | 0.06 | 0.81 | 0.11 | 0.64 | 0.02 | 0.93 | -0.05 | 0.82 |
|  | Glu/Cr | -0.08 | 0.74 | -0.16 | 0.50 | -0.01 | 0.97 | -0.03 | 0.88 |
|  | Glx/Cr | -0.22 | 0.34 | -0.18 | 0.43 | -0.11 | 0.63 | -0.24 | 0.29 |
| Left hippocampus | [^11^C]UCB-J DVR | -0.16 | 0.49 | -0.04 | 0.85 | -0.03 | 0.90 | -0.31 | 0.18 |
|  | Glu/Cr | -0.28 | 0.22 | -0.19 | 0.40 | -0.13 | 0.56 | -0.22 | 0.34 |
|  | Glx/Cr | -0.29 | 0.21 | -0.37 | 0.10 | -0.11 | 0.63 | -0.14 | 0.55 |

1. Institute of Clinical Sciences (ICS), Faculty of Medicine, Imperial College London, London W12 0NN, UK. [↑](#footnote-ref-1)
2. Psychiatric Imaging Group, Medical Research Council, London Institute of Medical Sciences, Hammersmith Hospital, London W12 0NN, UK. [↑](#footnote-ref-2)
3. Department of Psychosis Studies, Institute of Psychiatry, Psychology & Neuroscience, King’s College London, London SE5 8AF, UK. [↑](#footnote-ref-3)
4. Centre for Psychiatry and Mental Health, Wolfson Institute of Population Health, Queen Mary University of London, London E1 2AB, UK. [↑](#footnote-ref-4)
5. East London NHS Foundation Trust, 9 Alie St, London E1 8DE [↑](#footnote-ref-5)
6. City & Hackney Early and Quick Intervention in Psychosis, East London NHS Foundation Trust, London, UK [↑](#footnote-ref-6)
7. South London and Maudsley NHS Foundation Trust, Camberwell, London SE5 8AF, UK. [↑](#footnote-ref-7)
8. IQVIA [↑](#footnote-ref-8)
9. Department of Psychological Medicine, Institute of Psychiatry, Psychology, and Neuroscience, King’s College, London, UK [↑](#footnote-ref-9)
10. Tower Hamlets Early Intervention Service, 51 Three Colts Lane, Bethnal Green, East London NHS Foundation Trust, London, E2 6BF [↑](#footnote-ref-10)
11. Department of Brain Sciences, Imperial College London, The Commonwealth Building, Hammersmith Hospital, Du Cane Road, London W12 0NN, UK. [↑](#footnote-ref-11)
12. Research and Development Department, West London NHS Trust, London, United Kingdom [↑](#footnote-ref-12)
13. Lakeside Unit, West Middlesex University Hospital, West London NHS Trust, Twickenham Road, Isleworth, London TW7 6AF [↑](#footnote-ref-13)
14. Westminster Community Rehabilitation Team & Bluebell Lodge, Central and North West London NHS Foundation Trust, 7A Woodfield Road, London W9 2NW [↑](#footnote-ref-14)
15. Kensington Chelsea and Westminster Early Intervention Service, Central and North West London NHS Foundation Trust, Hathaway House, 7F Woodfield Road, London W9 2BA [↑](#footnote-ref-15)
16. Invicro, Burlington Danes Building, Du Cane Road, London W12 0NN, UK. [↑](#footnote-ref-16)
17. Centre for Neuroimaging Sciences, Institute of Psychiatry, Psychology and Neuroscience, King’s College London, De Crespigny Park, London SE5 8AF, UK. [↑](#footnote-ref-17)
